# How does gut microbiota affect the vaginitis axis? The mediating role of plasma metabolites

**DOI:** 10.1128/spectrum.02263-24

**Published:** 2024-12-31

**Authors:** Mo Li, Qianyu Zhang, Tong Wu, Lanfang Ma, Dianxing Hu, Zixuan Yuan, Shixuan Wang, Aiyue Luo, Jinjin Zhang

**Affiliations:** 1 Department of Obstetrics and Gynecology, Tongji Hospital, Tongji Medical College, Huazhong University of Science and Technology, Wuhan, Hubei, China; 2 National Clinical Research Center for Obstetrical and Gynecological Diseases, Wuhan, Hubei, China; 3 Department of Obstetrics and Gynecology, Guiyang Maternity and Child Health Care Hospital, Guizhou, China; Chengdu University, Chengdu, Sichuan, China

**Keywords:** Gut microbiota, Vaginitis, Plasma metabolites, Mendelian randomization, Causal relationship, Mediation effects

## Abstract

Vaginitis is the most common problem afflicting women of childbearing age. However, the underlying etiological factors remain poorly understood, leading to recurrent vaginitis and constraining clinical management. Here, we explored whether the gut microbiota influences the risk of vaginitis by performing a two-sample Mendelian randomization analysis using the largest genome-wide association studies to date. Four gut taxa in genus levels were identified related to vaginitis: *Candidatus Soleaferrea* (inverse-variance weighted [IVW] odds ratio [OR] = 2.20, *P* = 0.026), *Dialister* (IVW OR = 2.62, *P* = 0.029), *Lachnospiraceae UCG-008* (IVW OR = 0.41, *P* = 0.0067), and *Ruminiclostridium 5* (IVW OR = 0.080, *P* = 1.42 × 10^−5^). We further explored the mediation effect of the plasma metabolites by two-step Mendelian randomization (MR) and multivariable MR. The findings indicated that 3-phosphoglycerate and lysophosphatidylcholine antagonistically act against the two identified risk factors (*Candidatus Soleaferrea* and *Dialister*, respectively) of vaginitis, thus appearing to confer protective effects against vaginitis. On the contrary, the elevation of arachidonate/pyruvate ratio and reduction in palmitate/myristate ratio mediated the protective effects of *Lachnospiraceae UCG-008* against vaginitis. These findings support a potential causal role for gut microbiota in the development of vaginitis, thereby providing potential strategies for its prevention and intervention.

**IMPORTANCE:**

Vaginitis is the most common problem afflicting women of childbearing age. However, the underlying etiological factors remain poorly understood, leading to recurrent vaginitis and constraining clinical management. Besides, the human gut and vagina are important organs that are both colonized by thousands of microorganisms impacting human physiology and health. Whether there is an interplay between the microecosystems is intriguing and unclear. This study evaluated the potential causal relationship between the gut microbiota and vaginitis and suggested that specific types of gut microbiota may be the potential risk or protective factors of vaginitis mediated or suppressed by certain plasma metabolites. These findings provide treatment insights for vaginitis.

## INTRODUCTION

Vaginitis is one of the most common complaints of women through all stages of life (1), accompanied by symptoms of vulvovaginal itching, irritation, burning, odorous, abnormal vaginal discharge, etc. The prevalence of vaginitis is estimated over 50% in Asian and African American women, and a 29% overall prevalence of bacterial vaginosis (BV), which is the most common type of vaginitis, has been reported (1–3). Vaginitis is a heterogeneous condition characterized by diverse clinical manifestations, with BV, vulvovaginal candidiasis, and trichomonas being the most prevalent types (4). Disturbance in the vaginal microbial environment can lead to severe obstetrical and gynecological issues, such as adverse pregnancy outcomes (5), preterm labor (6), low conception rates (7), post-partum endometritis (7), and pelvic inflammatory disease (8). However, the etiology of vaginitis is still unknown. Although the treatment of vaginitis is considered straightforward, treatment failure is common, and the recurrence is up to 30% within 3 months and 58% within 12 months (9). Therefore, identifying the risk factors of vaginitis is vital in developing prevention and treatment strategies.

The gut microbiota (GM) has been proven to be a vital part of the human body affecting health, including the female reproductive system (10). Observational studies have found associations between gut microbiota and polycystic ovarian syndrome (11), endometriosis (12), and gynecologic cancers (13), which may be linked to their influence on estrogen levels (14). Besides, unique groups of bacteria were detected in vaginal microbiome, which significantly influence reproductive and other processes. For example, a healthy vaginal microbiota can provide defense against external pathogens by activating local and systemic inflammatory responses. Additionally, the composition of the vaginal microbiome can impact *in vitro* fertilization outcomes, neonatal immune function, and metabolic development and may be associated with the risk of spontaneous preterm birth (15–17). Recent studies indicate that complex correlations may exist between the gut and vaginal microbiota (18–22). For example, the origin of the cervicovaginal bacteria has been traced to the rectum (20), and a strong correspondence between the vaginal and rectum bacteria loads has been observed (21). Crosstalk between microorganisms in the gut and vagina is possible both horizontally, from the gut to the vagina within the same individual, and vertically, from the mother to the child (22). Given these hints, the GM may contribute to or resist the progress of vaginitis. However, there is barely any study that investigates the causal relationship between GM and vaginitis. Notably, the GM is one of the major contributors to the circulating pool of small-molecule metabolites of the hosts. Whether the gut and vaginal microbiota may communicate with each other through the circulating metabolites remains unclear.

Mendelian randomization (MR) is an ingenious method to infer the causal relationships between modifiable exposures and different outcomes. Based on Mendel’s law of inheritance, alleles are randomly assorted from parents to offspring. MR uses genetic variants and single-nucleotide polymorphisms (SNPs), called instrumental variables (IVs), as the basis for grouping to simulate the randomized controlled studies. With this underlying hypothesis, MR can circumvent biases from confounding factors and reverse causality in traditional observational studies (23, 24). In this study, we utilize the previously published summary-level genetic association data to infer the potential causal relationships between GM and vaginitis and reveal the mediation effect of the plasma metabolites by MR analysis. Our findings uncover the underlying risk or protective factors of vaginitis, dissect the potential mechanisms of how GM affects the outcome of vaginitis, and provide possible prevention and treatment strategies against vaginitis.

## MATERIALS AND METHODS

### Study design and data sources

**Fig 1:**
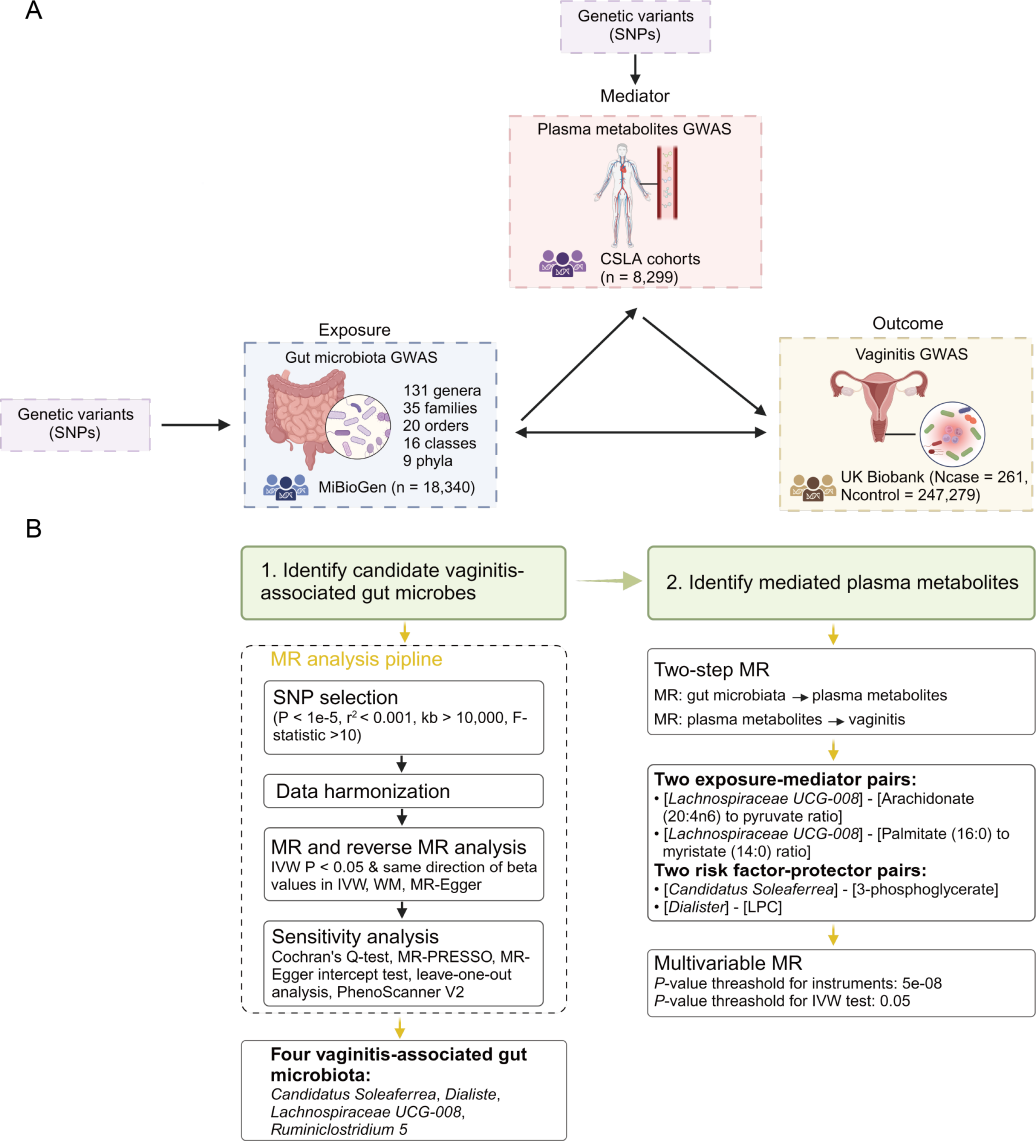
Study overview and analysis pipeline. (A) Overview of the study design. (B) Pipeline of the two-sample MR analysis and the mediation analysis. Figures are created with BioRender.com.

The overall study design is illustrated in Fig. 1. We used the GM as the continuous variable exposure and vaginitis as the binary outcome to inspect the relationship between them by a two-sample MR analysis. Besides, the mediation effects of plasma metabolites were evaluated by two methods: two-step MR and multivariable MR (MVMR) (Fig. 1A and B). The two-sample MR requires the data of the summary-level genome-wide association studies (GWAS) of both exposures and outcomes. The GM GWAS data come from the MiBioGen consortium (25). They meta-analyzed GWAS data of the GM from 24 population-level cohorts include 18,340 individuals from the United States, Canada, Israel, South Korea, Germany, Denmark, the Netherlands, Belgium, Sweden, Finland, and the United Kingdom. Among these cohorts, 20 cohorts include samples from single ancestry: 16 cohorts of European (*n* = 13,226), one cohort of Middle Eastern (*n* = 481), one cohort of American Hispanic/Latin (*n* = 1,097), and one cohort of African American (*n* = 114). The remaining four cohorts comprise samples from multiple ancestries (*n* = 2,571). The MiBioGen consortium curated the genome association data for 211 gut taxa in total. After removing unknown taxa, 196 taxa were included in this study, including nine phylum-level taxa, 16 class-level taxa, 20 order-level taxa, 35 family-level taxa, and 131 genus-level taxa.

The GWAS summary statistics of vaginitis were obtained from the GWAS Catalog Database (ID: GCST90044303) (26), which includes 261 European ancestry cases and 247,279 European ancestry controls from the UK Biobank (UKB) resource. The definition of vaginitis is based on the *International Classification of Disease* (ICD), 10th edition, and then mapped to Phecode 614.52. The genetic data were genotyped by the Applied Biosystems UK Biobank Axiom Array and the Applied Biosystems UK BiLEVE Axiom Array (27). The SNPs were imputed using the whole-genome sequence data from the Haplotype Reference Consortium (28) and the UK10K project (29) as reference panels. The data were filtered with the standard quality control (QC) criteria in PLINK2 (30). More details for QC were described in the original paper (26). After QC, 11,842,647 variants were imputed. Next, the fastGWA-GLMM method (26) was used to perform the genome-wide association analysis, and age, age^2^, and the top 20 principal components provided by the UKB were fitted as covariates.

The GWAS summary data of plasma metabolite come from the GWAS study of the Canadian Longitudinal Study on Aging (CLSA) cohort, including 8,299 unrelated individuals of European ancestry (31). Details of the cohort information and GWAS analysis can be found in the original studies (31, 32). Briefly, genotyping was done using the Trans-Omics for Precision Medicine program (33) and genetic ancestry determination by the CLSA group (34). QC of the SNPs included the following standard: (1) minor allele frequency (MAF) higher than 0.1%, (2) imputation quality score > 0.3, and (3) missing rate < 0.1, leading to approximately 15.4 million SNPs (in reference build 38) for GWAS testing. The 1,400 metabolites were quantified using the ultrahigh performance liquid chromatography–tandem mass spectroscopy platform. After strict QC (31), GWAS was performed using linear regression of the metabolites and metabolite ratios by the fastWGA tool from GCTA version 1.93.2 beta (35).

As only the publicly available summary-level data were used in the present study, no additional ethical approval or participant consent was required.

### Instrumental variables

Three core IV assumptions must be satisfied in the MR analysis: the IVs are strongly related to the exposure; the IVs share no unmeasured cause with the outcome; and the IVs do not affect the outcome, except through their potential effect on the exposure of interest. Therefore, the following criteria are applied in this study to guarantee that the core IV assumptions are met: (1) the *P*-values of associations between SNPs and taxa less than 1 × 10^−5^ were retained as potential IVs, as in previous studies (36–38); (2) to avoid linkage disequilibrium (LD) between SNPs, we performed LD clumping using the reference panel data from the 1000 Genomes project (European samples); clumping r^2^ was set as 0.001, and the window size was set as 10,000 kb; (3) the SNPs should not be associated with the outcome; therefore, only SNPs with association *P*-values > 1 × 10^−5^ in the outcome GWAS data are retained; and (4) to assure the intensity of each IV, we calculated the F-statistic, and only SNPs with F-statistic > 10 were retained. The F-statistics were calculated by the following equations:


$$\begin{array}{l} F=\;\left(\frac{R^{2}}{1-R^{2}}\right)\left(\frac{n-k-1}{n}\right)\\ R^{2}=2\times \left(1-MAF\right)\;\times MAF\;\times \;\beta^{2} \end{array}$$

in which *R*^2^ represents the variance explained by each SNP; *n* is the sample size; and *k* is the number of selected IVs (39, 40). To avoid potential pleiotropy, PhenoScanner V2 (40) was used to detect SNPs correlated with any risk factors of the vaginitis.

### Two sample MR analysis

For exposures that have more than one SNP as IVs, we performed the MR analysis based on three methods: inverse-variance weighted (IVW) method, weighted median (WM) method, and MR-Egger method using the “TwoSampleMR” package (41). The three methods independently inferred the associations between each taxon and the outcome. Since the IVW method has superior power under certain conditions (42), the results of IVW were used as the predominant criteria for causal inference. The other two methods were also important references. Taxa with the IVW *P*-value < 0.05 and all the effect values (β) calculated by three methods being in the same direction were considered significant results associated with the outcome. If the exposure has only one SNP as IV, the Wald test was performed to test the association between the exposure and vaginitis. The criterion *P* < 0.05 was used to select significant associations. Besides, the *P*-values were adjusted by Bonferroni correction, in which the *P*-values were multiplied by the number of comparisons.

### Sensitivity analysis

Cochran’s Q-test was used to assess the heterogeneity of IVs from different studies (43). A Q-statistic *P* < 0.05 indicates that heterogeneity exists in the MR analysis results. Besides, MR-pleiotropy residual sum and outliers (MR-PRESSO) was used to detect outliers of SNPs that indicate horizontal pleiotropy. If outliers were detected in the MR-PRESSO global test, they were removed from the IVs, and MR analyses were re-conducted. The MR-Egger intercept test is used to assess whether the SNPs have pleiotropic effects on the outcome. *P* > 0.05 indicates the intercept differing from zero is not significant, and there is no horizontal pleiotropy. Lastly, the leave-one-out analyses were conducted to assess the effect of each SNP on the outcome.

The pleiotropy of SNPs was also evaluated by first examining whether the SNPs were associated with other significant exposures (GM or metabolites). These multi-traits correlated with SNPs were removed. Then, we searched the PhenoScanner database to see if SNPs selected as IVs were correlated to vaginitis. If genetic variants were detected at risk of horizontal pleiotropy, we removed these SNPs and repeated the MR test.

### Mediation analysis

There are two strategies to perform mediation analysis. One is the two-step MR (or network MR), which separately performs univariable MR between the exposure and the outcome, the exposure and the mediator, and the mediator and the outcome (44). In this study, in addition to the primary effect of the GM on vaginitis (β, total effect), we also estimated the effects of the GM on the plasma metabolites (α) and the plasma metabolites on vaginitis (β_2_) following the same MR analysis procedures. The indirect effect defined as the effect of one gut microbe acting on vaginitis through one mediator plasma metabolite is estimated by α × β_2_.

The other strategy for mediation analysis is the MVMR method, in which the gut microbe and the plasma metabolite are both treated as exposures and regressed against the outcome of vaginitis. The coefficient between gut microbe and vaginitis is the direct effect (β_1_). The indirect effect is calculated as β − β_1_. In both strategies, the proportion of mediated effects is estimated by the indirect effect/total effect.

## RESULTS

### Study overview

We used two-sample MR to investigate the relationship between GM and vaginitis and the potential mediation effects of plasma metabolites. The overall study design, including data source, and population information, was illustrated in Fig. 1A, Table 1, and the Methods section. The pipeline of the univariable MR and mediation analyses, including both two-step MR and MVMR, is shown in Fig. 1B.

**TABLE 1:** Information of summary-level GWAS data used for MR analysis

Exposure/outcome	Data source	Sample size	Ethnic origin	Download	PMID
Gut microbiota (119 genera, 32 families, 20 orders, 16 classes, and nine phyla)	MiBioGen consortium	18,340	European, Hispanic, Middle Eastern, Asian, and African	https://mibiogen.gcc.rug.nl/menu/main/home/	33462485
Plasma metabolites	GWAS catalog (ID: GCST90199621-90201020	8,299	European	https://www.ebi.ac.uk/gwas/publications/36635386	36635386
Vaginitis and vulvovaginitis	GWAS catalog (ID: GCST90044303)	261/247,279 (case/control)	European	http://ftp.ebi.ac.uk/pub/databases/gwas/summary_statistics/GCST90044001-GCST90045000/GCST90044303/	34737426

### Genetic correlation between GM and vaginitis

**Fig 2:**
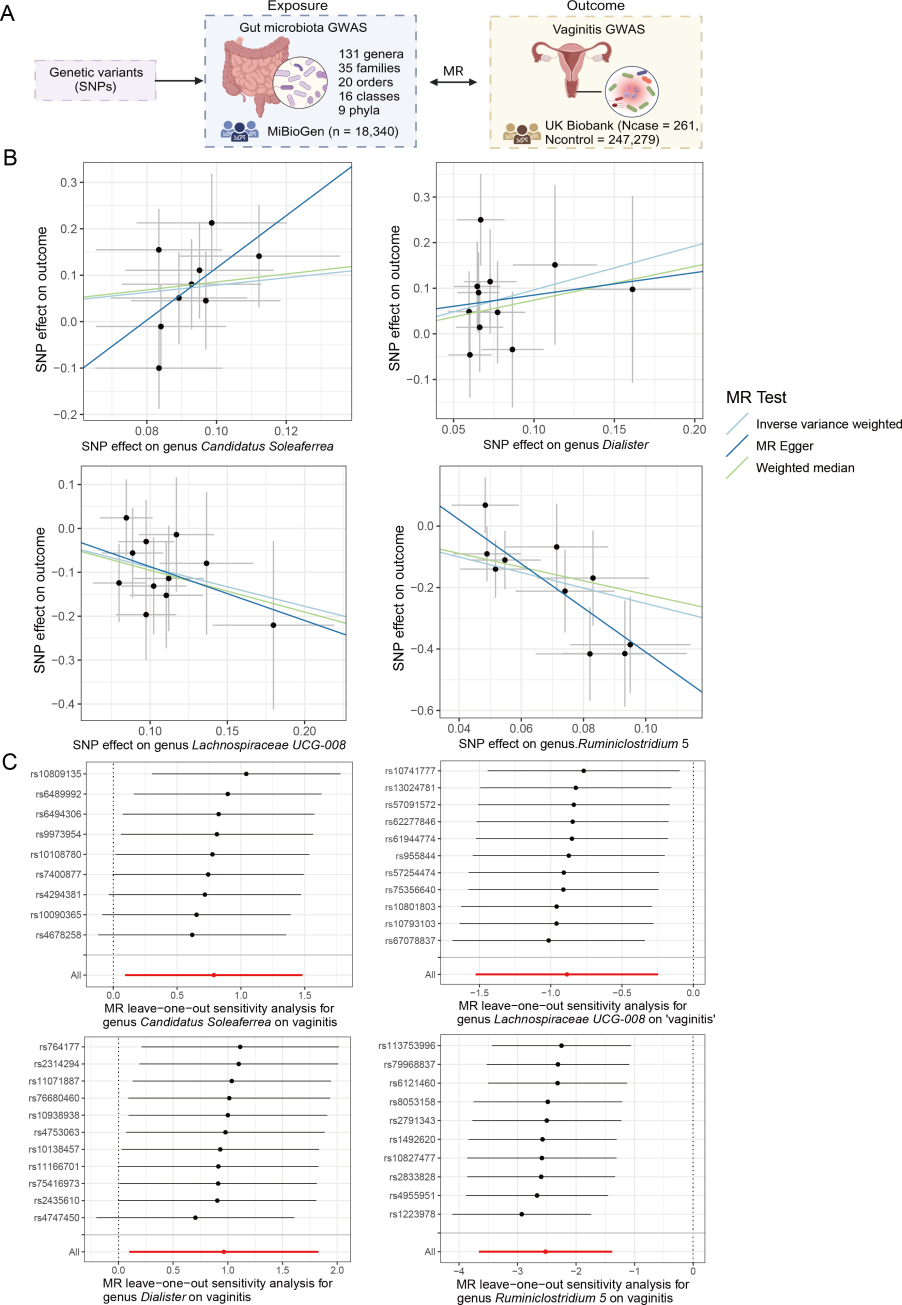
Associations between four taxa and vaginitis by Mendelian randomization (MR) analysis. (A) A schematic diagram showing the bidirectional MR analysis between the gut microbiota (GM) and vaginitis. (B) Scatterplot showing the associations between four GM taxa (*Candidatus Soleaferrea*, *Dialister*, *Lachnospiraceae UCG-008*, and *Ruminiclostridium 5*) and vaginitis. (C) Forest plot of the leave-one-out analysis. Each single-nucleotide polymorphism (SNP) was removed for each calculation of the effects of GM on vaginitis using the rest of the SNPs. The red line indicates the β value and the 95% confidence interval calculated by all SNPs.

We first investigated the potential causal relationship between the GM and vaginitis (Fig. 2A). For each of the 196 GM taxa, the correlated SNPs from the MiBioGen GWAS were filtered by *P* < 1 × 10^−5^, LD r^2^ < 0.001, and window size > 10,000 kb. This filtration resulted in 2,578 SNPs in total, and details of each SNP and taxa are listed in Table S1. The risk factors of vaginitis include cigarette smoking, under-managed diabetes, treatment with antibiotics, high-estrogen contraceptives, thyroid or endocrine disorders, sexual activity, douching, characteristics of menstruation, and menstrual product use (45). Among these risk factors, phenoScanner V2 detected two SNPs (rs6494306 and rs10458299) associated with type 2 diabetes and self-reported parathyroid gland problems (Table S2). These two SNPs were removed subsequently to avoid pleiotropy of the MR analysis. After further quality control and data harmonization (Methods), 2,102 SNPs were used as IVs for the following MR analysis. The numbers of SNPs used as IVs for each taxon ranged from 3 to 21 (median 11, Table S3). The F statistics of the IVs were all above 10, ranging from 12.8 to 155.5 (median, 57.34, Table S3), ensuring no weak instrument bias existed.

### Two-sample MR analysis between GM and vaginitis

Based on the results of three MR methods, namely, IVW, weighted median, and MR-Egger, we found that four taxa at the genus level were associated with vaginitis. Specifically, two genera were positively correlated with vaginitis risk: *Candidatus Soleaferrea* (IVW OR = 2.20, 95% confidence interval (CI): 1.10–4.40, *P* = 0.026), *Dialister* (IVW OR = 2.62, 95% CI: 1.10–6.23, *P* = 0.029). Two genera were negatively correlated with vaginitis risk: *Lachnospiraceae UCG-008* (IVW OR = 0.41, 95% CI: 0.22–0.78, *P* = 0.0067), *Ruminiclostridium 5* (IVW OR = 0.080, 95% CI: 0.026–0.25, *P* = 1.42 × 10^−5^) (Fig. 2B). All these taxa had the same correlation direction in the three methods. Notably, *Ruminiclostridium five* was the most significant genus (Bonferroni-adjusted *P* = 0.0028) because the IVW *P* value was the smallest, and all *P* values were below 0.05 in three methods (Table 2). The comprehensive MR results of all taxa are shown in Table S4.

**TABLE 2:** Significant GM taxa associated with vaginitis

Exposure (gut microbiota)	Method	N (SNP)	Beta	SE	*P*-value	OR	95% CI
*Candidatus Soleaferrea*	IVW	9	0.788	0.355	2.63E−02	2.198	1.097–4.404
*Candidatus Soleaferrea*	Weighted median	9	0.855	0.495	8.40E−02	2.351	0.892–6.2
*Candidatus Soleaferrea*	MR Egger	9	5.629	3.846	1.87E−01	278.377	0.148–523208.49
*Dialister*	IVW	11	0.964	0.441	2.90E−02	2.622	1.104–6.227
*Dialister*	Weighted median	11	0.74	0.587	2.08E−01	2.097	0.663–6.631
*Dialister*	MR Egger	11	0.492	1.789	7.89E−01	1.635	0.049–54.464
*Lachnospiraceae UCG-008*	IVW	11	−0.887	0.327	6.66E−03	0.412	0.217–0.782
*Lachnospiraceae UCG-008*	Weighted median	11	−0.953	0.429	2.65E−02	0.386	0.166–0.895
*Lachnospiraceae UCG-008*	MR Egger	11	−1.225	1.68	4.84E−01	0.294	0.011–7.906
*Ruminiclostridium 5*	IVW	10	−2.521	0.581	1.42E−05	0.08	0.026–0.251
*Ruminiclostridium 5*	Weighted median	10	−2.228	0.802	5.47E−03	0.108	0.022–0.519
*Ruminiclostridium 5*	MR Egger	10	−7.182	2.315	1.46E−02	0.001	0–0.071

The reliability of these significant MR results was further confirmed by sensitivity analyses. The results from Cochran’s Q-test showed that there was no heterogeneity among the selected SNPs, as all Cochran’s *P*-values were larger than 0.05 (Table S5). Besides, there were no significant outliers in SNPs detected by MR-PRESSO (Table S6). The MR-Egger intercept analysis further confirmed that no horizontal pleiotropy existed, as the intercept *P*-values were all above 0.05 (Table S7). The leave-one-out analyses revealed that no single SNP significantly influenced the correlations between *Lachnospiraceae UCG-008*, *Ruminiclostridium 5*, and vaginitis (Fig. 2C). However, for *Candidatus Soleaferrea* and *Dialister*, the exclusion of three SNPs and one SNP, respectively, may potentially affect the significance of the MR analyses (Fig. 2C), warranting further verification. Overall, the leave-one-out analyses demonstrated the robustness of the MR analysis results.

We next examined the reverse relationship using vaginitis as exposure and each GM taxa as the outcome. No SNP was left if we set the *P*-value threshold as 5 × 10^−8^; thus, we utilized the same standard as the initial MR when selecting IVs (*P* < 1 × 10^−5^, LD r^2^ < 0.001, window size > 10,000 kb). A total of 19 independent SNPs without LD were selected as IVs for the MR analysis (Table S8). We followed the same pipeline as the initial MR analysis. The family *Bacteroidaceae* and genus *Bacteroides* were negatively affected by vaginitis, while the class *Clostridia*, order *Clostridiales*, genus *Adlercreutzia*, and *Eggerthella* were positively affected by vaginitis (Table S8). However, none of these taxa survived multiple testing corrections.

### Mediation analysis of plasma metabolites between the gut microbes and vaginitis

**Fig 3:**
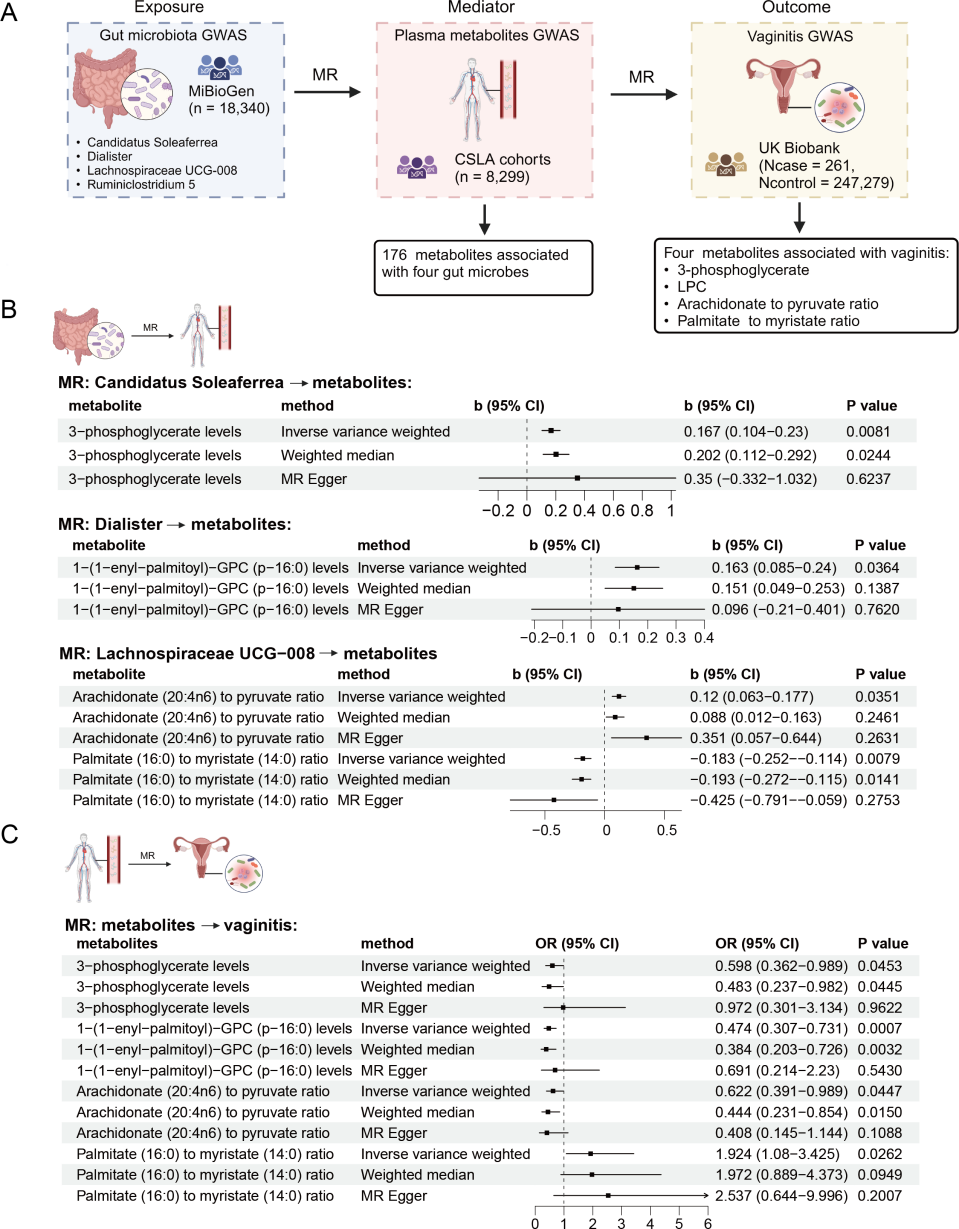
Two-step Mendelian randomization (MR) analysis of the mediation effects of plasma metabolites on the associations between gut microbiota (GM) and vaginitis. (A) A schematic diagram showing the data and results of the mediation analysis. (B) Significant results of the MR analysis between the screened GM taxa and four potential mediator plasma metabolites. (C) Significant results of the MR analysis between four potential mediator plasma metabolites and vaginitis.

Since one of the most prominent functions of the GM is to modulate the host metabolome, we hypothesize that the relationships between gut microbes and vaginal health are mediated by the plasma metabolites. Thus, we tested the mediation effects of the plasma metabolites between GM and vaginitis (Methods). We first performed the two-step MR analysis, including MR analysis from the exposures to the mediators and MR analysis from the mediators to vaginitis (Fig. 3A). Based on the four microbes we screened in the primary MR analysis, we performed the MR analysis between each pair of the four gut microbes and 1,400 plasma metabolites or ratios. A total of 176 plasma metabolites were significantly affected by one of the four gut microbes (Table S9; Fig. 3A), and two metabolites survived multiple testing corrections: 4-methoxyphenol sulfate (Bonferroni-adjusted *P* = 0.029) and salicylate to caprylate ratio (Bonferroni-adjusted *P* = 0.049), both related to the genus *Lachnospiraceae UCG-008*. To find more potential mediators between the gut microbiota and vaginitis, we continued subsequent analysis based on 176 metabolites.

**Fig 4:**
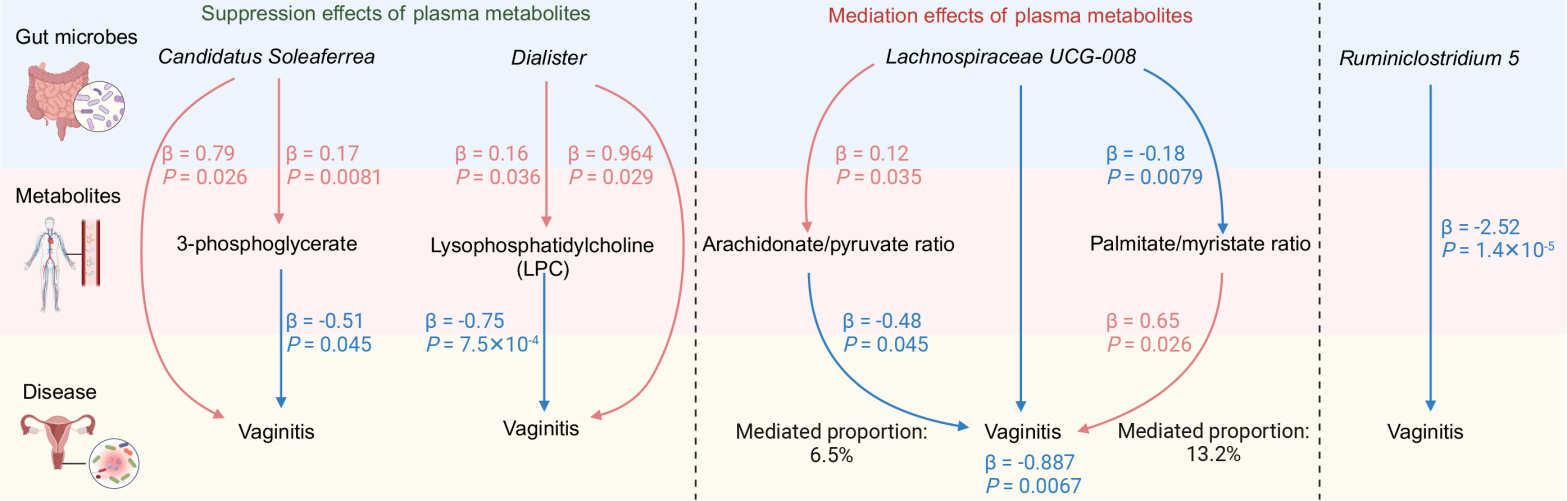
Summary diagram of the results of MR analysis between the GM and vaginitis, and the effects of plasma metabolites. The β values represent the effect estimates using the IVW method. Arrows and characters in red and blue colors indicate positive correlations and negative correlations, respectively. The mediated proportions of arachidonate/pyruvate ratio and palmitate/myristate ratio are calculated based on the method of two-step MR. If the directions of the two-step MR of metabolites agree with the direction of the initial MR from GM to vaginitis, the effects of metabolites are mediation. Otherwise, the effects are suppression.

To clarify whether these 176 metabolites were associated with vaginitis, we further performed an MR analysis between each metabolite and vaginitis (Table S10). Four metabolites were associated with GM and vaginitis at the same time (Fig. 3B and C). Genus *Candidatus Soleaferrea* was positively associated with 3-phosphoglycerate levels (IVW b = 0.167, se = 0.063, *P* = 0.0081), which were negatively associated with vaginitis (IVW OR = 0.598, 95% CI: 0.362–0.989, *P* = 0.0453). Genus *Dialister* was positively associated with the level of 1-(1-enyl-palmitoyl)-GPC (p-16:0), known as lysophosphatidylcholine (LPC) (IVW b = 0.163, se = 0.078, *P* = 0.036), which were negatively associated with vaginitis (IVW OR = 0.598, 95% CI: 0.362–0.989, *P* = 0.0007). However, the mediation effects of these two metabolites were opposite to the primary MR results of *Candidatus Soleaferrea* and *Dialister* with vaginitis, both of which were positive associations indicating the suppression effect of these two metabolites (Fig. 4). In comparison, the genus *Lachnospiraceae UCG-008* was positively associated with the arachidonate-to-pyruvate ratio (IVW b = 0.12, se = 0.057, *P* = 0.035), which was negatively associated with vaginitis (IVW OR = 0.622, 95% CI: 0.391–0.989, *P* = 0.045). *Lachnospiraceae UCG-008* was also negatively associated with palmitate-to-myristate ratio (IVW b = −0.183, se = 0.057, *P* = 0.0079), which was positively associated with vaginitis (IVW OR = 1.924, 95% CI: 1.08–3.425, *P* = 0.026). The directions of the mediation effects of the two metabolites were consistent with the direction of the primary *Lachnospiraceae UCG-008* effect on vaginitis (negative association), indicating the mediation effect of the two metabolite ratios between *Lachnospiraceae UCG-008* and vaginitis. The proportions of the mediation effects of the arachidonate-to-pyruvate ratio and the palmitate-to-myristate ratio were estimated to be 6.5% and 13.2% (Fig. 4), respectively.

To further confirm the effects of the four plasma metabolites on the mediation between gut microbes and vaginitis, we performed MVMR analyses. We fitted the independent MVMR models for each of the four pairs of potential exposure-–mediator pairs. The adjusted direct effects of each exposure on the vaginitis showed the same direction as the effects estimated by univariable MR analysis (Table S11). These results also showed suppression effects of 3-phosphoglycerate and LPC levels on the association between *Candidatus Soleaferrea, Dialister,* and vaginitis, respectively, and mediation effects of arachidonate-to-pyruvate ratio and palmitate-to-myristate ratio on the association between *Lachnospiraceae UCG-008* and vaginitis, with mediated proportions of 14.1% (*P* = 8.2 × 10^−4^) and 16.5% (*P* = 0.024), respectively. Together, these results indicated that certain plasma metabolites can mediate the effects between the beneficial GM and vaginitis, or act as protective factors against the detrimental gut microbes to the vagina.

## DISCUSSION

Vaginitis is one of the most prevalent female complaints whose etiology remains unclear. In this study, we explored the potential causal relationship between GM and vaginitis by IVW, WM, and MR-Egger methods and identified four genera associated with vaginitis. Among them, *Candidatus Soleaferrea* and *Dialister* are positively correlated with vaginitis, and *Lachnospiraceae UCG-008* and *Ruminiclostridium 5* are negatively correlated with vaginitis, verified by multiple sensitivity analysis, including Cochran’s Q-test, MR-PRESSO global test, MR-Egger intercept analysis, and leave-one-out analysis. We further explored how these GM taxa affected vaginitis by mediation of plasma metabolites. The results suggested that two metabolites, 3-phosphoglycerate, and LPC, showed suppressive effects against the promotion effects of the *Candidatus Soleaferrea* and *Dialister* on vaginitis, respectively. *Lachnospiraceae UCG-008* exerted protective effects against vaginitis by increasing the ratio of arachidonate to pyruvate and decreasing the ratio of palmitate to myristate.

*Candidatus Soleaferrea* has been reported as a benign bacterium that exerts an anti-inflammatory effect in the gut (46). The correlation between *Candidatus Soleaferrea* and vaginitis has not been studied, but MR analyses have shown that this bacterium has a negative relationship with type 2 diabetes (47) and female infertility (48). Type 2 diabetes is a risk factor for vaginitis, and female infertility is closely related to vaginitis, which may explain the association between *Candidatus Soleaferrea* and these diseases.

Besides, we explored whether plasma metabolites mediated the association. Notably, while 3-phosphoglycerate was associated with both the abundance of the gut bacterium *Candidatus Soleaferrea* and the incidence of vaginitis, the directions of these correlations were contradictory to the direct correlation between *Candidatus Soleaferrea* and vaginitis. This suggests that the effects of *Candidatus Soleaferrea* on vaginitis were likely exerted through mechanisms other than the plasma metabolites. It is possible that 3-phosphoglycerate functions as an antagonist in response to the increased abundance of *Candidatus Soleaferrea* in the gut, as the elevation of *Candidatus Soleaferrea* abundance is associated with increased plasma 3-phosphoglycerate levels, which potentially inhibits the progression of vaginitis and thereby serving as a protective factor of vaginitis. 3-phosphoglycerate plays an important role in the energy metabolism. Through glycolysis, 3-phosphoglycerate can generate lactic acid, which is the major molecule that keeps the vagina in a low PH state (49). Thus, 3-phosphoglycerate might benefit the vagina through the generation of lactic acid.

*Dialister* is a common bacteria in the human gut that produces acetate and propionate (50). However, studies have reported that the high abundance of *Dialister* in the vagina is associated with BV (51, 52). Our MR analysis further showed that the *Dialister* in the gut may also contribute to the higher risk of vaginitis. Since the anus is the closest organ to the vulva, the anus can serve as an extravaginal reservoir of vaginal bacteria and increase the risk of incident BV (53). Therefore, the association between *Dialister* and vaginitis may result from the auto-transfer of *Dialister* from the anus to the vagina through hygiene practices or sexual activity. Besides, *Dialister* in the gut may affect the vaginal bacteria through secondary metabolites (54). We tested whether any plasma metabolites mediated the association between *Dialister* and vaginitis. The results indicated that the mechanisms by which gut *Dialister* affects vaginitis are not mediated through plasma metabolites. However, the metabolite LPCs may exert an antagonistical effect on the increase of the gut *Dialister*, potentially safeguarding the vagina against vaginitis. Multiple studies have indicated the anti-inflammatory role of LPCs at the vessel-–endothelial interface (55), and decreased levels of LPCs were associated with multiple physical dysfunctions, such as diabetes (56), polycystic ovary syndrome (57), rheumatoid arthritis (58), aging (59), etc. This result might indicate an antergic and protective effect of LPC against the promotion effect of *Dialister* on vaginitis.

*Lachnospiraceae UCG-008* belongs to the *Lachnospiraceae* family, *Firmicutes* phylum, and benefits the gut and human health by producing short-chain fatty acid (SCFA) (60). Our analysis suggested a protective effect of *Lachnospiraceae UCG-008* on vaginitis, which could be partially mediated by the elevated level of arachidonate-to-pyruvate ratio and decreased level of palmitate-to-myristate ratio. Free arachidonate and metabolites can promote and modulate type 2 immune response, helping resist infection of pathogens (61). Pyruvate is the end-product of glycolysis and an ingredient for ATP production. The lower arachidonate to pyruvate ratio indicates increased fatty acid metabolism secondary to decreased glucose usage, which has been characterized by diabetic cardiomyopathy (62). This mechanism may also explain the protective effect of arachidonate to pyruvate ratio on vaginitis. On the other hand, palmitate and myristate are both long-chain saturated fatty acids, whose dysregulation is closely related to health-related problems. Palmitate has been suggested to be able to induce inflammation in multiple tissues (63–65). The myristoylation of proteins, the process of which adds myristate to proteins by N-myristoyltransferase (myristoyl-CoA), is essential for protein localization and many biological functions. A higher level of myristate may indicate a higher level of protein myristoylation. Meanwhile, palmitoyl-CoA can competitively inhibit the myristoyl-CoA enzyme (66). However, these pieces of evidence did not demonstrate direct associations with vaginitis. Therefore, further research is necessary to elucidate the biological mechanisms underlying the relationships among *Lachnospiraceae UCG-008*, metabolites, and vaginitis.

*Ruminiclostridium 5* is the most significant taxa associated with vaginitis, though we did not screen any plasma metabolite that may mediate or suppress the association. *Ruminiclostridium 5* has been suggested to benefit the circadian rhythm and recovery from sleep fragmentation (67, 68). Besides, a low abundance of *Ruminiclostridium 5* is associated with acute pancreatitis and is reported in patients with kidney stones (69). However, the extent to which these associations reflect causal relationships between *Ruminiclostridium 5* and vaginitis remains uncertain. Further research is required to elucidate the mechanisms underlying the association.

In the reverse MR analysis, it was observed that the family *Bacteroidaceae* and the genus *Bacteroides* exhibited a negative association with vaginitis, whereas the class *Clostridia*, order *Clostridiales*, and genera *Adlercreutzia* and *Eggerthella* demonstrated positive associations. Studies have indicated that dysbiosis in vaginal microbiota, such as BV, can provoke a similar inflammatory phenotype in the gut (20). For example, *G. vaginalis* infection of the vagina can stimulate systemic inflammation and lipopolysaccharide production, resulting in increased *Proteobacteria* to *Bacteroidetes* and *Firmicute* to *Bacteroidetes* ratios (70). The findings of this report were consistent with our results that *Bacteroidaceae* and *Bacteroides* were negatively associated with vaginitis. Therefore, vaginitis may contribute to gut dysbiosis through mechanisms involving systemic inflammation.

The major strength of this study is that we linked the gut microbes with the health of vagina with MR analysis and explored potential mechanisms for the first time. These results are supported by the largest genome-wide association studies corresponding to each phenotype and robust sensitivity analysis. However, there are several limitations of this study. The GWAS studies used in this study are mostly European population cohorts, which may restrict the application of the conclusions in other populations. Also, the relationship between gut microbiota and vaginitis, and the mediation effects of plasma metabolites inferred in this study are results from computational analysis based on large cohorts of populations, and validation of the experimental studies is needed to make the conclusions more solid. Moreover, the associations with vaginitis identified in this study cannot be differentiated among specific types of vaginitis. Given that the GWAS study was conducted on broadly classified vaginitis, the definite gut microbes that are associated with each specific type of vaginitis have yet to be determined. Lastly, certain risk factors for vaginitis, such as sexual activity, intravaginal practices (e.g., douching), menstrual characteristics, and menstrual product use, were not evaluated due to the absence of GWAS data. These factors may affect the accuracy of the MR analysis results.

In summary, our study reveals the possible causal effects of gut microbes on vaginitis and identifies plasma metabolites as potential mediators or suppressors. *Candidatus Soleaferrea* and *Dialister* in the gut are risk factors for vaginitis. Their effects are suppressed by plasma metabolites 3-phosphoglycerate and lysophosphatidylcholine, respectively. *Lachnospiraceae UCG-008* is a protector for vaginitis mediated by increased arachidonate/pyruvate ratio and decreased palmitate/myristate ratio. *Ruminiclostridium 5* is also a protector of vaginitis, with no mediating or suppressing metabolites identified. Future studies are needed to verify the potential causal or protective role of each gut microbes and metabolite in animal studies and human samples. This study may provide useful insights into the future therapeutic targets of vaginitis.

## Data Availability

The source summary-level GWAS data can be accessed through MiBioGen consortium, GWAS Catalog (ID: GCST90199621-90201020), and GWAS Catalog (ID: GCST90044303). All the analyzed results data are available in the article table or supplementary materials.

## SUPPLEMENTAL MATERIAL

Tables S1 to S7, S9, and S11 spectrum.02263-24-s0001.xlsx This file includes supplementary tables of SNP information of the exposures, harmonised data, results of MR analyses, sensitivity analyses, and mediation analyses.

Table S8 spectrum.02263-24-s0002.xlsx SNPs of vaginitis used as IVs in the reverse MR analysis.

Table S10 spectrum.02263-24-s0003.xlsx Harmonized data for MR analysis between each of the 176 plasma metabolites and vaginitis.

ASM does not own the copyrights to Supplemental Material that may be linked to, or accessed through, an article. The authors have granted ASM a non-exclusive, world-wide license to publish the Supplemental Material files. Please contact the corresponding author directly for reuse.
